# Shifting Perspectives: A Community-Based Learning Science Outreach Course That Engages Undergraduate Metacognition through Midsemester Redesign

**DOI:** 10.1021/acs.jchemed.4c00656

**Published:** 2025-03-17

**Authors:** L. Gaby Avila-Bront

**Affiliations:** Department of Chemistry, College of the Holy Cross, 1 College St., Worcester, Massachusetts 01610, United States

**Keywords:** Service Learning, Community-Based Learning, Chemistry Outreach, Metacognition

## Abstract

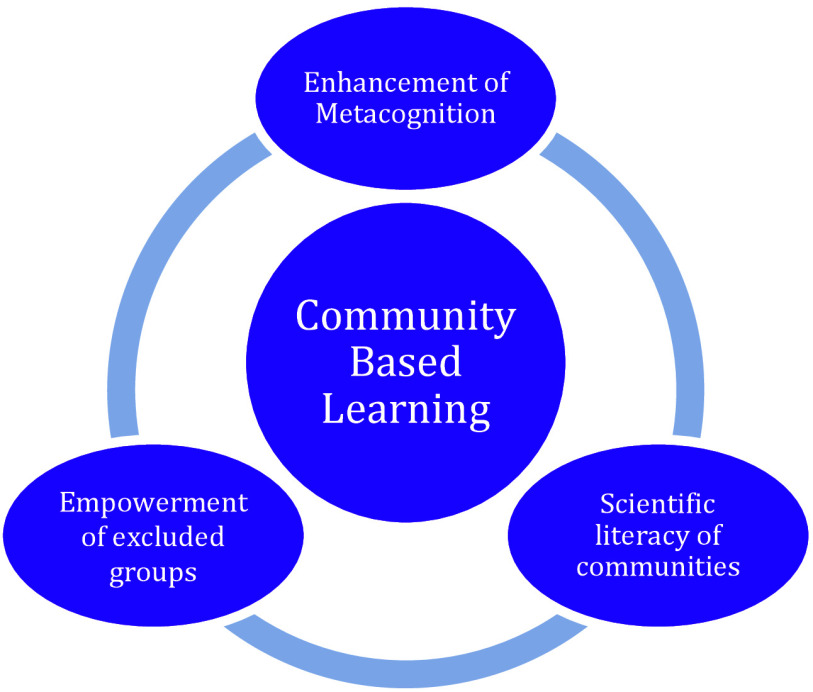

The content and implementation of a one-credit course with built-in science outreach to the community partner, Girls, Inc., is described herein. This community-based learning (CBL) course aims at developing the undergraduates’ ability to plan, monitor, evaluate, and adjust to new learning environments by reflecting on their own learning and also to define their scientific identities. The course delivery is divided into five Training and Preparation sessions, seven Community Partner Interaction sessions, and two Assessment sessions. Throughout the semester, the participating students reflected on their experiences and contextualized the outreach within the educational theories discussed. Student feedback indicated a shift in the undergraduate students’ bias on gender in science, and the *appreciation* to have the opportunity for metacognitive reflection.

## Introduction

It is well established that there is a pressing need to develop metacognitive skills in undergraduate students.^1^ Traditional chemistry courses rely heavily on the delivery content rather than maintaining a balance between content and development of metacognitive skills.^2^ Regardless of the scientific discipline, many undergraduate students enter higher education with inadequate experience in self-regulated learning, a crucial component of metacognition.^3^ This gap in pedagogical strategies manifests itself in the students’ struggle to identify effective learning strategies, which leads to limited adjustment of their comprehension of course material, and low exam preparedness.^4^ In college environments that demand greater independence compared to high school, these shortcomings can lead to poor academic performance and increased student stress. Enhancing metacognitive skills through targeted interventions like reflective practice sessions and metacognitive training workshops can significantly improve students’ ability to engage with and succeed in their college coursework.^5^ The development of metacognitive skills would also solidify the transfer of scientific inquiry skills regardless of students’ postcollege paths to enable them to identify and solve scientific problems.^6^

The metacognitive pedagogical gap described above exist on the backdrop of broader educational challenges such as the scientific literacy of communities,^7,8^ the interest of young students in science, technology, engineering, and mathematics (STEM) fields,^9^ the widening achievements gaps of students in quantitative reasoning skills^10−14^ and the representation of minority groups and women in STEM fields.^15−17^ Science educators around the country have proposed and launched a myriad of programs that address each of these issues and work toward a common solution.^18^ Though there are several successful programs in place at the College of the Holey Cross and similar institutions^11^ to address the challenges mentioned above, it is well-known that students require attention to these issues at earlier developmental stages.^19,20^

The course described herein gave undergraduate students the opportunity to both address the educational challenges stated above, and develop their metacognitive skills in the context of a mentor/mentee relationship with an elementary school student. This course engaged a local community partner and included a Community Based Learning (CBL, also known as Service Learning) component. Publications describing courses in scientific disciplines that implement CBL are a burgeoning field.^21−24^ In two examples, courses at Tulane University and the University of Arizona worked with the undergraduate and/or graduate students to develop outreach content while also providing training on community engagement.^25,26^

Through its Jesuit mission and policies, the College of the Holey Cross (Worcester, MA) emphasizes the benefit of weaving community service into the tapestry of education by having an established office dedicated to CBL.^27−29^ In the 2022–2023 academic year, the Donelan Office for Community Based Learning sustained 48 courses that combined classical education with CBL.^30^ The mission of the office is to engage students, faculty, and community partners in “teaching, learning, and engaged research that address pressing community issues.” The benefits of CBL to student learning are apparent in feedback from students and community partners. Over 90.8% of the 783 students who participated in that year reported that “including CBL in this course enabled me to learn more deeply than I otherwise would have.” In addition, 95.7% of the community partners stated that “Hosting students was worth the time and energy required from my agency’s staff”. Based on the research conducted by the National Survey of Student Engagement, CBL significantly and positively impacts student engagement and success.^31,32^ This can be explained by the fact that CBL can lead to “great accommodation”, i.e., the point of largest change in cognitive development, described by Love and Guthrie, when a student shifts from perceiving the world as understandable to recognizing its intricate complexities.^33^

## Community Partner

One of the roles of the Donelan Office is to identify community partners and match them to the courses teaching with CBL components. The match is made based on the availability of the community partner and the needs of the specific course. *Girls, Inc.*, a national nonprofit organization that provides programming such as after school clubs and summer camps for young girls, was selected as the community partner for this course. During the semester, Holey Cross undergraduate students worked with girls between the ages of 7 and 10 who were attending the after school program at Girls, Inc. Reflective of the Worcester, MA community that it inhabits, many of the girls in this program come from mid to low socioeconomic households, and from diverse racial and ethnic backgrounds.^34^ Due to the demographics of the community partner, the lecture topics and primary literature focused on the participation of women in science, as well as the “leaky pipeline” of women in scientific fields. As an afterschool facility offering multiple programs, the Girls, Inc. staff presented their activities as “clubs” for which the girls would sign up to attend.

## Changing Course Goals to Match Needs of Community Partner

The original design of the course planned for undergraduate students to establish a mentor-mentee relationship with the elementary-aged students. Throughout the semester, the mentors and mentees would work on a science fair project according to a research question identified by the elementary school students.^35^ The undergraduate mentor students would develop metacognitive skills, such as planning, monitoring, evaluating, and adjusting by (1) creating mentoring sessions with the mentees, (2) preparing a plan of action in the shape of lesson plans for the mentoring session, and (3) developing a timeline for the science fair project. Concurrently, the younger students’ self-efficacy would benefit from a one-on-one mentoring relationship that would grow with time.^25,36^ However, we discovered that the after school programs at Girls, Inc. did not have regular attendees, and as a result, long-term science fair projects could not be pursued.

Subsequent sections of this manuscript will describe how pivoting the course created the opportunity for “challenges through cognitive dissonance” as described both by Chickering et al. and Sanford et al.^37−39^ Cognitive dissonance is the psychological discomfort or tension that arises when a person is confronted with conflicting beliefs, values, or attitudes. This discomfort often motivates individuals to resolve the inconsistency.^40^ These authors explain that while encountering cognitive dissonance can lead to significant growth in the students’ learning and development, it must be done in a supportive learning environment for positive change to occur. As a result of the cognitive dissonance, students’ experiences resulted in the simultaneous overlap of the steps of concrete experience, active experimentation, reflective observation and abstract conceptualization.^41^

## Course Structure

This course was taught for the first time in 2023 to eight students. Three students were in their fourth-year and five students were in their second-year. Although the class was offered in the chemistry department, the prerequisites for this course were any introductory chemistry, physics, neuroscience, or biology courses. Of the eight students that were enrolled, one was a physics major, one was a biology major, and the rest were chemistry majors. There were six cis-female students and 2 cis-male students.

The class met once a week in a time block of 2.5 h. The sessions were generally a combination of content delivered as PowerPoint slides, a class discussion of a primary literature article, and a visit to the community partner, Girls Inc. For class sessions that included a reading assignment, students read the journal articles in full before coming to class and came prepared to have a discussion with their peers. Once the site visits began, the first hour was reserved for a lecture/discussion, and then the undergraduate students traveled for approximately 30 min on a college-provided shuttle service or drove themselves to the location. At the site, the undergraduate students would have about 45 min to set up and complete the activity with the young students, and 10–15 min to clean up before returning to campus.

## Learning Blocks and Learning Objectives

The course was divided into three learning blocks. These learning blocks scaffolded the learning experience and progressed the undergraduate students’ learning through the cognitive, associative, and autonomous stages.^42−45^ Students were introduced to the theories supporting the learning objectives in the first learning block- *Training and Preparation*. In the second learning block, *Community Partner Interactions*, the learning objectives were put into practice as the students began the site visits to Girls, Inc. In the third learning block, *Assessment*, students reflected on and assessed their experiences. The learning objectives (LOs) specified in the syllabus are listed in Table 1. In addition, the table correlates each LO with the metacognitive skill(s) present in the implementation of the LO. Within this manuscript, the correlation of particular learning objectives to course content is referenced as “LO- #”. The metacognition of the LOs allowed for the LOs to remain the same throughout the semester, even after the course goals shifted.

**Table 1:** Learning Objectives as Listed in the Syllabus

Learning Objectives (LOs)		Correlated Metacognitive Skill(s)
LO-1	Become an active knowledge possessor of the scientific method by identifying a hypothesis, designing and experiment, and identifying variables.	Planning, Monitoring, Evaluating, Adjustment, Reflection
LO-2	Evaluate personal biases/cultural differences and how these affect student learning outcomes.	Evaluating, Reflection
LO-3	Become mindful of the role that misconceptions play in the learning process.	Monitoring, Evaluating, Reflection
LO-4	Create lesson learning objectives based on Bloom’s Taxonomy.	Planning, Monitoring, Evaluating
LO-5	Assess the level of Bloom’s Taxonomy within lesson objectives.	Planning, Evaluating, Adjustment,
LO-6	Develop valid assessment questions based on project and lesson objectives.	Planning, Monitoring, Evaluating, Adjustment,
LO-7	Improve your communication skills.	Monitoring, Evaluating, Adjustment, Reflection

### First Learning Block: Training and Preparation

The first learning block (Training and Preparation) set forth foundational pedagogical and metacognitive principles. Each lecture was accompanied by an in-class discussion of students’ reflections on their own learning values and challenges, and interpretations of the assigned readings. A full list of assigned readings and associated activities can be found in Table 2.

**Table 2:** First Learning Block

Week	Topic	Reading/Discussion/Assignment
1	Intro to CBL and Bloom’s Taxonomy	Discussion of course goals
2	Growth Mindset and Stereotype Threat	**Self-assessment**: Harvard IAT Gender-Science- Write reflection and share
		**Reading**: Growth-Mindset Intervention Delivered by Teachers Boosts Achievement in Early Adolescence, Psychological Science, 2022
3	Instrumentality	**Write and share**: Instrumentality Essay
		**Reading**: Who Leaves, Who Stays? Psychological Predictors of Undergraduate Chemistry Students’ Persistence, Journal of Chemical Education, 2015
4	Assessments	**Reading:** MA K-12 science curriculum
5	Learning misconceptions	Class discussion

In the first week, we established the vocabulary of pedagogy and learning according to Bloom’s Taxonomy. Once students have a pedagogical vocabulary established, they must work on their mindsets that all individuals are capable of learning and growing regardless of their backgrounds. As a result, in the second week, the students were introduced to Carol Dweck’s work on growth mindset^46^ by reading and discussing “Growth-Mindset Intervention Delivered by Teachers Boosts Achievement in Early Adolescence” by Porter et al.^47^ The lecture content also introduced stereotype threat.^48,49^ Prior to the lecture, students were tasked with confronting their own implicit biases by taking the Harvard Implicit Association Test (IAT) for Science and Gender (LO-2).^50^ During the class session, students shared their reflections on their IAT results. A full discussion of the results of the IATs is discussed in a subsequent manuscript section. By the third week, students were ready to define their personal goals for this course in the form of an *instrumentality* essay. Based on Vroom’s Expectancy Theory of Motivation,^51^ and similar to Bandura’s work on motivation and self-efficacy,^52^*instrumentality* surmises that individuals choose their behaviors based on what they believe leads to the most beneficial outcome. The prompt for this assignment is included in the Supporting Information. A summary of their personal goals for the course is described in the section of this manuscript on Assessment. The reading for this session was “Who Leaves, Who Stays? Psychological Predictors of Undergraduate Chemistry Students’ Persistence”, by Shedlosky-Shoemaker et al.^53^ We used this reading to discuss the students’ own motivation to persist in their science classes in the context of instrumentality and goal-setting. Students shared their own perceptions of introductory science classes as “weed out” courses for medical schools that made the environments difficult to thrive in as first-year undergraduates.

In the penultimate session of this first learning block, students crafted a set of assessment questions that they would give to their mentees as a pretest (LO-6). These assessments followed a class session on designing assessments using Bloom’s Taxonomy, and written summaries of the Massachusetts Department of Education Standards for K-6 education.^54^ As instructors, pretests can set the framework for expectations and provide an awareness of the students’ needs. Tasking students with creating pretests allowed them to define their priorities as teachers and learners. By basing their pretests on verbs from Bloom’s taxonomy, they reflected on which verbs were most important to them- e.g., the analysis verbs like comparing/contrasting or the memorization verbs like list/define. Many students designed pretests on the mentees’ motivation to learn science, which falls under the affective domain of Bloom’s Taxonomy. In the Assessment section of this manuscript, it will be discussed that several students discovered that motivation is decisive in learning science. To conclude the Training and Preparation learning block and to prepare for the start of the second learning block (Community Partner Interaction), we discussed misconceptions in learning (LO-3). Group discussions addressed students’ own misconceptions in learning, and how they worked through them. We brainstormed possible strategies to counteract common misconceptions in science learning as these ways of thinking might be present in the elementary students.^55^

Broadly speaking, the topics and discussions in the first learning block covered three learning objectives, and sought to begin clarifying students’ evolving identities as scientists. The class discussions encouraged students to share their ideas and built connections between the students and the faculty member. This learning block established the classroom ecology as a safe space for students to learn and share their experiences.

## Second Learning Block: Community Partner Interaction

In the second learning block, undergraduate students began the six-week site visits to the community partner. As aforementioned, the first hour of class sessions remained a lecture and discussion period, and the final 2 h were allotted for the site visit. Each week, students submitted written reflections on their experiences with the community partner and constructed a basic lesson plan for the next session with their mentee based on the interaction (LO-4). These writing assignments put into practice the metacognitive principles of planning, monitoring, evaluating, and adjusting. Table 3 outlines the topics, assignments, and site visit activities associated with this learning block.

**Table 3:** List of Session Topics and Corresponding Site Visit Activities in the Second Learning Block^a^

Week	Topics	Reading/Discussion/Assignment	Girls, Inc. Activity
6	What does it mean to *do* science?	**Reading:** Adolescents’ and Emerging Adults’ Implicit Attitudes about STEM Careers: “Science is Not Creative”, Science Education International, 2016	**Site Visit 1:** Assessments
7	*Who* can be a scientist?	**Discussion:** Discuss “Picture a Scientist” movie	**Site Visit 2:***Chaos!*
8	Change format of site visits		**Site Visit 3:** Marshmallow Skyscrapers
9	Discuss new course format	**Reading**: “Relational Scaffolding Enhances Children’s Understanding of Scientific Models” Association for Psychological Science, 2021	**Site Visit 4:** Making Slime
10	**Site visit 5**: Lava Lamps		
11	**Site visit 6**: Science Party		

a Due to various scheduling needs, sessions 10 and 11 were solely dedicated to site visits.

### Site Visit 1

To begin the second learning block, the theme of the first class session was “What does it mean to *do* science?”. In class, we discussed and critically reflected on the 2016 reading by S.S. Valenti et al.^56^ In this reading, Valenti et al. argue that many adolescents, especially females, did not view scientific careers as creative, and, as a result, did not wish to pursue careers in STEM fields. This reading was selected so that the students would be aware of their mentees’ possible perception of science. We revisited our discussion of implicit biases, and reinforced the second learning objective (Evaluate personal biases/cultural differences and how these affect student learning outcomes). Awareness of their bias would enable the students’ mindfulness in the discussions with their mentees and develop the students’ communication skills (LO-7).

At the site visit, the students got to know the mentees and administered the pretests. To provide a framework for their conversations, the students were equipped with a list of tentative learning objectives based on their personal goals and interests for the interaction. The complete prompt and a full list of the students’ learning objectives is included in the Supporting Information. The undergraduate students used their conversations with the mentees to discover the mentees’ scientific interests, and planned to return the following week with activities based on these interests to facilitate the mentees’ choice of a science fair project. To fill the remaining time in the session, the undergraduate students engaged the mentees with boxed engineering activities donated to Girls Inc. by the Boston Museum of Science.^57^

### Site Visit 2

Prior to the lecture and discussion portion of Site Visit 2, students watched the movie *Picture a Scientist*, and came to class prepared for a group discussion to address the week’s theme of “*Who* can be a scientist?”. *Picture a Scientist* is a 2020 documentary that examines gender inequality and discrimination within scientific communities. Throughout the group discussion, the students further clarified characteristics of their scientific identities via *prototyping*.^58,59^ We discussed how their scientific identities matched to the subjects in the documentary, and where they fit in in the dynamic of men and women in scientific fields.

Students proceeded to the site visit prepared with their earlier plan to engage with the mentees through the boxed engineering activities. However, upon arriving at the site, the undergraduate students discovered that almost none of the original attendees who had taken the assessments were present due to the drop-in nature of the after school attendance programming. As a result, the undergraduate students’ plans were completely dashed.

Comparative analysis of the undergraduate students’ submitted written reflections revealed clear thematic patterns, and a disheartened group of students. The prompt for reflections was open-ended; the students were asked to “reflect on the previous week’s experiences”. Each student reflection was unique and covered a variety of topics that were then grouped into themes. To illustrate these themes, Table 4 lists selected individual statements from the reflections that have been categorized into descriptive themes such as change of plans, mentee turnover, indifference of the mentee, or environmental chaos. It is important to note that the identified themes are rudimentary in nature. As the semester progressed, the thematic patterns present in the students’ written submissions became more complex.

**Table 4:** Statements from Students’ Written Reflections Categorized into Thematic Patterns^a^

Statement from student reflection	Theme(s)
*· “Unfortunately, I have not seen my mentee since week one, so this plan did not come to light.”*	Change of plans
	Turnover
*· “When I asked her if she liked science she said ‘no, it’s boring’.”*	Turnover
*· “Switching mentees was a little difficult because it resets expectations.”*	Indifference
*· “My student wasn’t at Girls, Inc. last week.”*	Turnover
*· “The prepared lesson is based on my mentee’s interest in light and electricity. But during our second meeting we were drawn to the engineering kit and an arts and crafts discussion”*	Change of plans
*· “The first full week after the initial testing week was a bit chaotic.”*	Chaotic
*· “Although the girl I gave the pretest to was not present, I want to incorporate her interest into an activity.”*	Turnover
*· “This week was chaotic at Girls, Inc.”*	Chaotic
*· “She didn’t seem interested in the ″scientific″ questions I was asking her about her flower, and she just wanted to keep drawing pictures.”*	Indifference
*· “Unfortunately, all of my plans flopped. The girls also didn’t seem motivated to ″do science″ and even told me this but we will persevere!”*	Change of plans
	Indifference

a Note that there are only 7 reflections because 1 student was absent.

### Site Visit 3

Upon entering the classroom before what would be their third site visit, the undergraduate students were even more exasperated than they alluded to in their reflection statements. One student stated *“we’re expected to teach them science, and they don’t even want to be there”* in a shaky voice. I invited them to enact the first learning objective, i.e., become an active knowledge possessor of the scientific method by identifying a hypothesis, designing an experiment, and identifying variables, and address this challenge using their skills in scientific inquiry. Rather than discussing the research article that was planned for that session, the students brainstormed how to address the challenges they were facing. In a single class session, they upended the format of the course in response to the community partner’s needs, and enacted metacognitive skills of planning, monitoring, evaluating, and adjusting.

The students identified that the sudden change of plans affected both themselves and the mentees. The disruption of their plans left the undergraduate students bewildered and paralyzed, unsure of what actions to take. Without clear objectives or tasks, the mentees became distracted from the undergraduate students, resulting in a disorganized and chaotic classroom environment. This environmental chaos led to mentee indifference. To manage the environmental chaos, the students chose to implement a single activity each week with the mentees. This approach would enable them to promptly engage the mentees and adapt to the attendance that week. However, this also meant that the undergraduate students’ assessment of the mentees could no longer be implemented, nor could the mentees pursue a long-term science fair project. Because the undergraduate students did not have multiple weeks to form a relationship with their mentee, each session would be even more important in reaching any learning objective set by the undergraduate students. This diminished the role of the undergraduate student as a deliverer of content, and empowered them rather to be facilitators of the mentee’s scientific inquiry. The short-term interactions also emphasized the importance and need for careful communication between the student and the mentees (LO-7), especially as it has been shown that student perception of instructors’ mindsets strongly correlates with student performance.^60^

**Figure 1:**
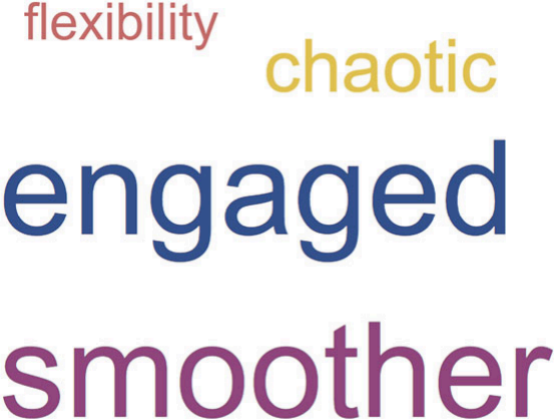
Qualitative analysis of student reflections in the form of a word cloud. Student perception of the third session show emerging themes in contrast to the difficulty expressed from the first two sessions.

The undergraduate students decided to spend the third site visit constructing marshmallow skyscrapers with the mentees. The thematic patterns that emerged from the students’ written reflections are shown in the form of a word cloud in Figure 1. The size of the word/phrase indicates the frequency of its occurrence. Once again, “chaos” became apparent as a theme. This time, however, the undergraduate students described the session as chaotic because the elementary students were so much more engaged and noisier as a result of their excitement to build marshmallow skyscrapers. The Supporting Information lists the students’ statements and how the statements were categorized (Table S1).

Pivoting the course’s design brought to light Howard’s writing on “principles of good practice” in CBL courses.^61,62^ His principles to “Rethink the faculty as a facilitator rather than a disseminator of content” and “Be prepared for variation, and some loss of control with student learning outcomes” fully characterized the undergraduate students’ experiences as they came to terms with the new nature of the course. Even as faculty members, this variation, loss of control, and role as a facilitator rather than a deliverer of content can be difficult to grapple with. Indeed, in a traditional lecture-based classroom or lab with upward of 50 students it can be impossible to do so. This experience and this course highlights the benefits and importance of having small groups of students as well as a decrease in content to allow for such flexibility–both of which were only possible with the format of this course.

### Site Visits 4–6

To begin Site Visit 4, students read “Relational Scaffolding Enhances Children’s Understanding of Scientific Models” by Jee et al., and engaged in a class discussion on this article.^63^ This study investigated the benefits of guided comparisons between observable and modeled events (relational scaffolding) to improve children’s comprehension of scientific models. During the in-class discussion, students associated relational scaffolding to their science laboratory courses. Students commented that their own learning was greatly improved when material in lecture was linked to experiments in the laboratory.

For the next three site visits, the undergraduate students split into groups of 2–3 and developed a single activity to conduct with the mentees. The groups were in charge of designing the learning objectives for the activity, purchasing supplies, testing the activity, and crafting any discussion questions or worksheets for the mentees. The activities for sessions 4–6 were: (4) making slime to discuss phases of matter, (5) creating lava lamps to discuss density, and (6) hosting a science party with activity stations that the mentees would rotate between. Class sessions for site visits 5 and 6 did not include a lecture portion due to scheduling logistics.

As the undergraduate students continued to reflect on their experiences, the qualitative analysis of their discussed themes showed a higher variation in themes and an increase in theme complexity. The thematic patterns showed that the undergraduate students delved deeper in their interactions with the mentees. Tables S2 and S3 in the Supporting Information show how the student statements were classified by theme. One of the new themes was the importance of the mentees to make predictions about the experiments before seeing any results in order to become invested in the outcome, putting into practice what they gleaned from the reading by Jee et al.^63^ However, several mentees became shy or unwilling to participate and expose themselves to the risk of being wrong. To respond to this, the undergraduate students encouraged a growth mindset by allowing the mentees to *freely* experiment with any materials provided. This led to an initial degree of “messiness” but also the greatest enthusiasm on the part of the mentees. The students asked the mentees guiding questions such as “what would happen if···” or “how can you prove me wrong”. Once the mentees were engaged with the active experimentation, their fear of “being wrong” diminished. This showed a development of mindset in both the undergraduate students and the mentees. In their written reflections and classroom discussions, the undergraduate students cited these moments as the most beneficial to the mentees and to themselves. The students described seeing their mentees’ faces light up or their speech become more fast-paced and excited. Simultaneously, the undergraduate students became cognizant of their role as facilitators to create an encouraging and participatory learning environment, where the learning itself lay in the hands of the mentees. The submitted written reflections and class discussions revealed that the undergraduate students had ironed out many of the kinks that were discovered earlier. The shift in the foci of the undergraduates to “*intrigue the girls’ curiosity*” and “*keep girls engaged*” through “*predictions and experimentation*” reveal an implementation of metacognitive skills by the undergraduate students.

## Learning Block 3: Assessments

The third and final learning block focused on assessment (Table 5). All assessment activities were reviewed by the Institutional Review Board (IRB) at the College of the Holey Cross, and were exempt from informed consent. Students also consented that their assignments could be shared. The readings for the 12th and 13th sessions were “The brain adapts to dishonesty” and “The acquisition of the gender-brilliance stereotype: Age trajectory, relation to parents’ stereotypes, and intersections with race/ethnicity”.^64,65^ Both readings further contextualized the shift in students’ attitudes toward the course and biases toward science and gender as they prepared for their final reflections. Active discussion of the students’ shifting perspectives, raised their awareness that a change in mindset was occurring.

**Table 5:** Topics and Reading Assignments in the Third Learning Block: Assessment

Week	Topic	Reading
12	Neuroplasticity	“The brain adapts to dishonesty” Nature Neuro 2016
*Academic Conference*–College-wide presentations of independent student work.		
13	Assessments/Reflections	“The acquisition of the gender-brilliance stereotype: Age trajectory, relation to parents’ stereotypes, and intersections with race/ethnicity.” Child Development, 2022
		**Assignment**: Learning Gain Essay

In addition to (1) reflecting on their learning gains throughout the semester and comparing these gains with the goals they identified in their Instrumentality Essays, students (2) filled out in-class surveys, and (3) retook the Harvard IAT and reflected on their new score. A brief discussion and the results of the IATs is included in the Supporting Information.

### (1) Instrumentality and Learning Gains Essays

At the end of the semester, the students wrote a “Learning Gains” essay, which prompted them to “review the goals submitted in the Instrumentality Essay, and reflect on how they have changed, and what [the students] have learned.” The full prompt is available in the Supporting Information. The comparison between statements from the students’ Instrumentality Essays (Learning Block 1), and statements from the students’ Learning Gains Essays (Learning Block 3) are summarized in Table S5 of the Supporting Information. Though the precise language used by each student varies, it is possible to group the statements into thematic patterns. Based on the Instrumentality Essays, the most common goal was development of the students’ communication and/or mentoring skills. However, when drawing meaning from the Learning Gains Essays, multiple themes are present, and it becomes difficult to color code the students’ reflections due to the complexity and intersectionality of the thematic patterns.

The thematic patterns of the Learning Gains Essays demonstrate an implementation of the following metacognitive skills: planning, monitoring, evaluating, and adjusting. The practicing of these metacognitive skills is evident in students’ references to a growth mindset, flexibility, communication, and the importance of motivation in studying science. To adopt a growth mindset, students must first be flexible in their thinking to adjust to changing scenarios. With a growth mindset established, students develop their communication skills to address the needs of the mentees. In the Learning Gain Essays, the theme of “communication skills” is mentioned in the context of *connecting* with their mentee by adopting a student-centered approach to learning. Being cognizant of their mentees’ needs, students mention “*careful communication*” and being “*mindful and aware*” of the words that they are using when engaging with mentees. This demonstrates a transformation of the theme of communication from a mere concept to a component of a relationship. Simultaneously, students work to define their scientific identity by deciding who they want to be as science communicators, and what they prioritize in learning environments. For example, some students discuss fostering curiosity, while others focus on encouraging a growth mindset, and still others emphasize hands-on activities.

### (2) In-Class Surveys

At the end of the semester, the students completed two surveys. One was designed and administered by the faculty member (Supporting Information), and the second was designed and administered by the Donelan Office for CBL (discussed below).

## CBL Survey

**Figure 2:**
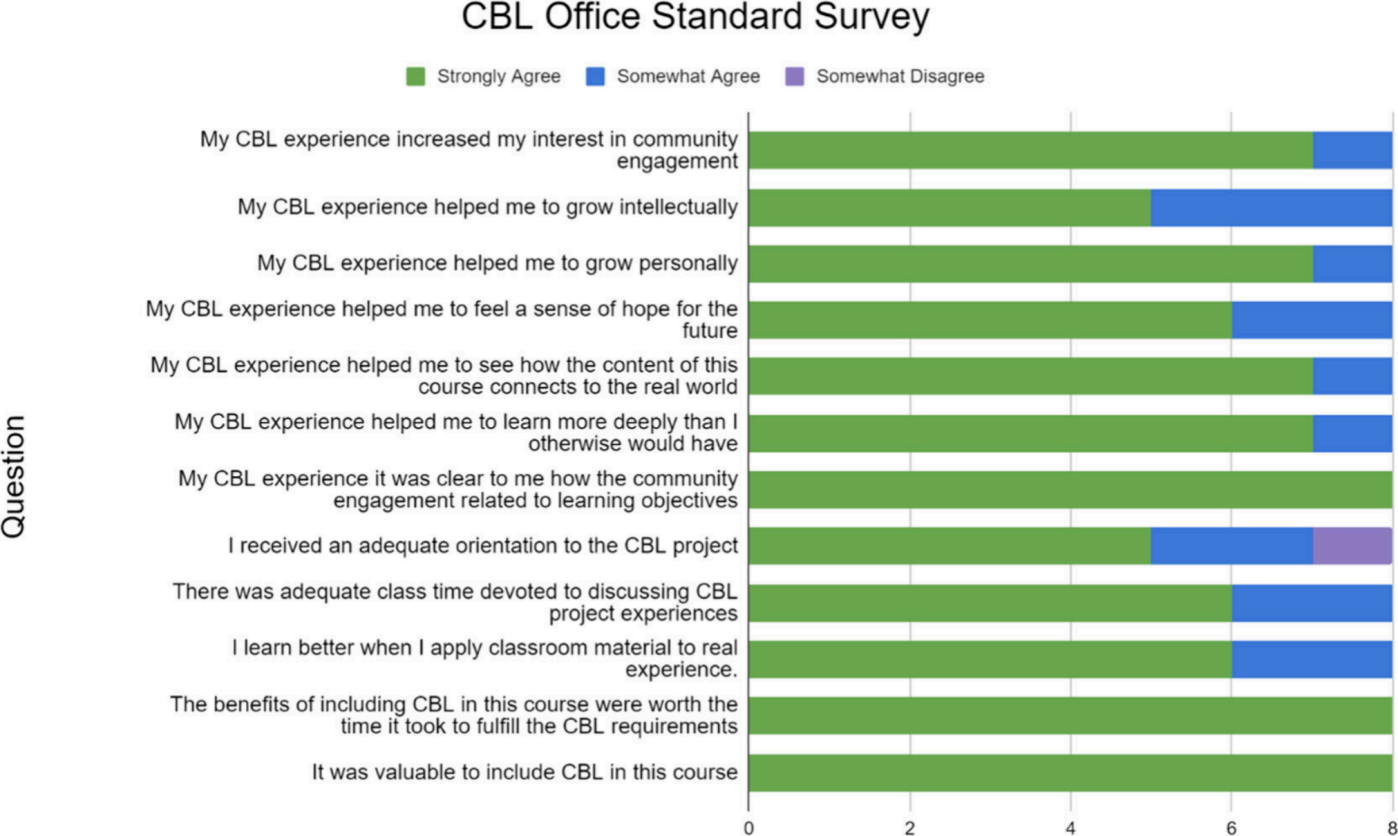
Results of the survey administered by the Donelan Office for CBL. Survey results demonstrate the students’ appreciation for CBL in a STEM context.

Results of the survey administered by the Donelan Office for CBL for this specific course are shown in Figure 2. The purpose of this survey is to ascertain the effect that the CBL experience has on student learning. The students responded anonymously and completed the survey in-person during the final class session. Responses to the survey indicated that the students greatly appreciated working in the community. All the students strongly agreed that the benefits of CBL in the course were worth the time it took to fulfill the CBL requirements, and that it was valuable to include CBL in the course.

## Conclusion

The course described herein exposes various angles of learning, such as individual assessment of strengths and weaknesses, commitment to the community needs, and development of a new and creative perspectives of flexibility in the mentoring process. Analysis of undergraduate students’ written reflections, as well as class discussions, show that CBL is a favorable arena for contextual understanding of STEM identity and the development of metacognitive skills of the undergraduate students. Written reflections and class discussions convey how students processed and transferred skills during active experimentations with their mentees to resolve the ‘cognitive dissonance’ that they were experiencing at the start of the course. This resolution resulted in their motivation to continue their CBL learning objectives, adaptation to the mentees’ needs, and deeper awareness of their scientific identities. CBL courses can help strengthen students’ STEM identity and develop their metacognitive skills by applying classroom concepts and engaging in discussions about personal purpose, values, the role of systems, and how to effectively collaborate in learning environments.

## Supporting Information Available

Learning Block 1: Learning objectives assignment prompt; submitted student work: learning goals for their mentees and pretests; instrumentality essay assignment prompt; weekly writing assignment prompt. Learning Block 2: Statements from student reflections sorted into thematic patterns. Learning Block 3: Assessment assignment prompts selected; statements from students’ learning gains essays; student reflections from learning gains essays compared to goals identified in instrumentality essay; results from the faculty-administered survey; results from students’ IATs from the beginning and end of the semester; statements from student reflections on their end-of-semester IAT results (PDF, DOCX)
