# Supplementary material for: Shifting Perspectives: A Community-Based Learning Science Outreach Course That Engages Undergraduate Metacognition through Midsemester Redesign
Source: J Chem Educ. 2025 Mar 17;102(4):1436–44. doi: 10.1021/acs.jchemed.4c00656 (PMC11984092; doi:10.1021/acs.jchemed.4c00656)
Supplement: Supplementary file 2 — ed4c00656_si_002.docx [file ed4c00656_si_002.docx]

Shifting Perspectives: A Community-Based Learning Science Outreach Course that Engages Undergraduate Metacognition through Midsemester Redesign

**L. Gaby Avila-Bront***

*Department of Chemistry, College of the Holy Cross*

*1 College St. Worcester, MA, 01610, USA*

* To whom correspondence should be addressed.

*E-mail address: lavila@holycross.edu*

**Learning Block 1: Training and Preparation**

LEARNING OBJECTIVES PROMPT AND STUDENT RESPONSES

**Learning Objectives Assignment Prompt:**

The assessment that you will deliver to your students on your first meeting will be based on the learning objectives that you define for the program. These learning objectives are entirely based on your own priorities and values. Thus, it is up to you if you want your students to learn facts, gain a sense of belonging, or become more curious about their world. Your assessment will reflect the values that you set out.

To complete this assignment:

1. Define several learning goals- anywhere between 5 and 10 should be a good amount.
2. Create a set of assessment questions - again, between 5 and 10 questions- that assess these learning objectives.
3. To gain an understanding of what kind of science background your students have, read your assigned grade level in the document “MA DoE Standards” (posted to Canvas). Create a 1 page summary of the specific grade level knowledge that you can expect your students to have. Please email me. Consult the table below to know which grade level you are responsible for reading. Post your summary in our class Canvas page under “Discussions”

**Learning Goals**

1. Students will be able to identify the steps of the scientific method

2. Students will be able to apply the steps of the scientific method to investigate problems

of interest to them.

3. Students will be able to defend scientific claims through the collection of data

4. Students will be able to design an experiment to test problems of interest

5. Students will be able to compare observations before and after an experimental test to

understand what occurred

**Learning Objectives:**

Student will be able to observe an experiment and retell their understanding by creating a set of

written observations.

Student will be able to classify their observations and takeaways from an experiment by

categorizing and sorting them into known groups.

Student will be able to communicate their observations and categories of an experiment to their

classmates.

Student will be able to measure and gather data from observations or experiments.

Student will be able to infer and theorize on the experiment based on their findings and data.

Student will be able to predict outcomes of the same or similar experiments based on their

findings.

Student will be able to design and innovate new experiments based on their experience with the

prior investigation.

**Learning Objectives:**

Focus: Confidence is key!

By the time of the science fair, students will be able to…

1. Feel more confident in their knowledge of a topic in science they chose to explore

2. Have the confidence to explore further topics in science they may be interested in on their

own

3. Feel more confident as a young girl interested in pursuing STEM overall, regardless of

the field potentially being male-dominated

4. Apply skills in problem-solving and critical-thinking taught throughout our time together

to all aspects of life, in and out of the classroom

5. Pose questions of topics they are unsure of in both large and small group settings,

regardless of the gender of those present

**Learning Goals**

● Expand their definition of what a scientist “is”, who can be a scientist, and what a

scientist does

● Increase interest in science, math, and engineering

● Become more self-confident in their abilities in math and science

● Be able to define a science fair and a sample science fair project

● Be able to define how an experiment is created and walk through the scientific method

**My Learning Goal:**

I want students to understand what STEM is, find what subject in STEM they like the most, and

be able to recognize that science is all around them.

**Learning Objectives:**

Students will be able to identify the different aspects of STEM

Students will be able to identify what area of STEM they enjoy the most

Students will be able to list multiple reasons why this is their favorite subject

Students will gain a deeper understanding of their chosen area of STEM

Students will gain an appreciation for the variety of options in STEM

Students will recognize STEM in their daily life

**Learning objectives:**

1. I want to increase students’ curiosity about the science around them.

2. I want students to be able to understand the science behind their chosen science fair topic and be

able to teach others about it.

3. I want students to develop the skill of asking intriguing questions.

4. I want students to collaborate with each other and build upon existing knowledge.

5. I want students to be able to apply their knowledge to explain other related ideas.

6. I want students to feel comfortable making mistakes and confident in learning from them.

7. I want students to have fun!

**LEARNING OBJECTIVES:**

1. Students will be able to form hypotheses surrounding their own observations
2. Students will be able to relate the scientific concepts they learn to real-world scenarios
3. Students will be able to make predictions about outcomes of new situations based on previous observations and knowledge
4. Students will gain confidence in trying to answer their own questions before asking teacher/chaperone for help
5. Students will be able to identify the independent and dependent variables in experiments.

**Learning Objectives:**

1. Students will be able to list the steps of the scientific method in order.

2. Students will be able to define and provide examples of each step of the scientific method.

3. Students will be able to determine whether or not a question is able to be answered by the

scientific method.

4. Students will be able to assess solutions and chose the most feasible solution to a problem.

5. Students will be able to use the scientific method to design and perform experiments.

**Instrumentality Essay Assignment Prompt:**

In the 1980s, educational psychologists began to measure the importance of an individual’s understanding of the incentive for a present behavior. Studies defined this understanding as “task value” and “instrumentality”. One educational model/ theory states that the perceived value of a task is dependent on the individual’s understanding of “the contingent nature of each of the steps and the perceived value of the long-term goal and the steps leading to that goal” (Contemporary Educational Psychology 29 (2004) 63–76). As such, the purpose of this assignment is to encourage you as a student to identify and reflect upon your goals in the CBL aspect of this course.

To complete this assignment:

Write an essay identifying and reflecting on your personal goals related to the CBL component of this course. Your essay should be 650 words and should cover the following:

What CBL means to you

Why you were interested in this course

How CBL is related to your larger goals as an individual / student

How each aspect of this course can act as a stepping stone towards your larger goals (use the Syllabus/ Assignments/ Course readings to guide this discussion)

Come to class prepared to discuss your goals.

**Weekly Writing Assignment Prompt:**

Due to the open-ended nature of your meetings, these weekly writing assignments are flexible. Each week, please be sure to include (1) a reflection on how the previous meeting proceeded, (2) any adjustments to future meetings based on these reflections, and (3) a “lesson plan” for the next meeting with your mentee.

**Learning Block 2: Community Partner Interaction**

| **Statement from student reflections on new course format** | **Theme(s)** |
| --- | --- |
| This past week, things went a lot smoother at Girls, inc. than in previous weeks. Everyone seemed to be much happier. Although it was a bit chaotic during class trying to come up with an activity for all the mentees to do, it all worked out in the end. | - Smoother |
| The last meeting went surprisingly well given the amount of time we planned the activity. We could tell the girls were excited by the setup we provided and they really enjoyed the candy aspect. | - Smoother - Engaged |
| Last week went smoother than previous weeks. We had great conversation and, dare I say, scientific discovery. | - Smoother - Engaged |
| During last week’s meeting, I found much comfort in our conversation as a class, and hearing other students’ perspectives on their experiences and what we should do going forward was a game-changer. We went in much more confident and goal-oriented. And our attitudes reflected very well on the mentees. They were much more engaged. | - Engaged |
| Last week at Girls Inc was very successful. Some girls stuck more closely to the science and engineering aspect than others, but all were learning and doing science even if they didn’t realize it! | - Engaged - Smoother |
| This week at Girls Inc. went much more smoothly with the group plan and activity. This gave a lot of flexibility with who the students work with, and allows for the chaotic and fun nature of the girls to not hinder the activity! | - Flexibility |
| This week was much better! It was still chaotic, but more in a “a bunch of young girls at the end of the day” type of chaos instead of “we have no idea what’s going on” type of chaos, which was easier to deal with. | - Chaotic - Engaged |

**Table S1:** A qualitative analysis of the themes of student responses regarding their reflections once the course format changed. This table lists out quotes from student essays and the themes in which they were categorized.

| **Statement from student reflections on the making slime activity** | **Theme(s)** |
| --- | --- |
| There was a consensus at the end of last week between us mentors that the written worksheets were not effective for the majority of the mentees. The adaptation and proper execution of a growth mindset could take us to the next level during our visits, from worksheets not being effective, to actually being able to pair the activities we are doing adequately with the science behind them. | - Careful communication - Growth mindset |
| Catrina’s batch was too liquidy and sticky. It didn’t look like some of the other girls to which she became disappointed. I asked her what she thought we could add to make it better. | - Discovery thru discussion - Experimentation |
| It got a little messy by the end! I had to direct the discussion about science because the girls were more interested in just playing with it but there was definitely some scientific discovery happening as they played with the slime. I think this is an excellent example of her learning from experiences and making connections between physical materials around her. | - Messy - Discovery thru discussion - Experiential learning |
| I found that discussing and walking through the worksheet with them was the best way to gauge their interest in the activity and teach them the science behind the slime. They seemed to respond better to open-ended questions where they could relate their interpretations and personal experiences. The final five minutes ended up being a bit crazy, with a lot of messes and cleaning up. | - Messy - Experiential learning - Discovery thru discussion - Bloom’s taxonomy |
| Last week was … MESSY. I will definitely keep in mind our work about “scientific misconceptions” in mind. I saw this firsthand when I couldn’t even identify the misinformation I found on the internet as a chemistry major, oops! | - Messy - Careful communication |
| The real learning and scientific probing came after the initial slime was made. She had no interest in writing, but talking through the worksheet really got her thinking. To investigate the phase of matter of slime, she began making slime leaving out certain aspects. | - Discovery thru discussion - Experimentation |
| While we didn’t answer the worksheet questions, we did problem solving and teaching. She decided to change what ingredients she added until she was happy with the result. | - Experimentation |

**Table S2:** A qualitative analysis of the themes of student reflections on the making slime activity. This table lists out quotes from student essays and the themes in which they were categorized.

| **Statement from student reflections on making lava lamps** | **Theme(s)** |
| --- | --- |
| I tried to remain asking questions to push the mentee I was working with to think about how the ingredients were mixing, but she was quite defiant and simply did not want to answer any questions! Unfortunately this does happen sometimes. | - Empathy |
| Something she said really stuck with me and it was, “I’m doing my own experiment!” | - Independent experimentation |
| Many students said the solutions would mix, while others said they would stay separate. | - Making predictions |
| Working with these girls breaks down barriers that I have made for myself and for other scientists, which has been helpful in creating a new definition of what it means to “do science”. | - Growth mindset |
| I thought of the many assets I had learned in class, mainly on the ability to foster a positive and fun environment in which the students would like to share their ideas and answer questions. | - Spark curiosity - Motivation |
| I made sure to probe the girls about why they predicted mixing or not mixing and this helped us engage in good scientific conversation. | - Making predictions - Independent experimentation |
| When she saw how the two layers separated, she was convinced that it was just because there was less oil, so I asked her to prove it to me. This challenge got her motivated.  She couldn’t figure out why that was happening, but she was so excited that she had created her own experiment. | - Motivation - Independent experimentation |

**Table S3:** A qualitative analysis of the themes of student responses regarding their reflections once the course format changed. This table lists out quotes from student essays and the themes in which they were categorized.

**Learning Block 3: Assessment**

**Assessment Assignment Prompts:**

1. Retake the implicit bias test to see if there has been any change and reflect on the results.
2. Revisit the goals that you submitted back in your *Instrumentality Essays*, and think about how you've changed and what you've learned. In terms of length- I'm wary of setting a particular length because I want the reflection to be organic. So rather than a word count, think about it this way- if someone were to look at your reflections at the start vs at the end of the semester, would they be able to have a thorough narrative?

| **Learning Gain Reflections** |
| --- |
| *“I had no idea that I would meet so many wonderful people, learn more about myself and the world around me than I could have ever imagined, or be inspired to pursue my passion for science even further. I had no idea that as I was trying my hardest to get young girls interested in science, that they would teach me more about myself than I was teaching them.”* |
| *“This year we have found a few major conclusions, STEM outreach to younger audiences need to revolve around fun. The stereotype kids gain where school, education, and homework isn’t fun is what kills any possibility of them listening to us “teach”. As soon as we bring science to them in a boring way, we lose their interest immediately. By creating fun, hands-on, interactive activities, the girls were more likely to listen to us and become curious about science. Another conclusion is we have to know our audience and tailor our approach to them. Many of the girls were easily distracted, as soon as another girl was doing something, they wanted to as well. In my experience with my mentee, there was no way that she was going to pay attention to a 5 minute video, nevermind continue with a 7 week long project. I think I learned when I figured out a new game plan going forward. Lastly, these meetings and this course can (and will) be largely unpredictable. The incoming mentors have to be open minded, problem solvers who can connect with many different personalities.”* |
| *“One of my goals that I wrote about in the Instrumentality essay is to improve my communication skills. I was originally writing this as improving science communication skills to a general audience, but I believe that this has expanded over the course of the semester. Communicating accolades, capturing the attention of young students, and maintaining focus were all important skills that I learned in this course that go beyond the science aspect. Most importantly, communicating that the students were doing well through their effort and hardwork was a shift in how I approached this experience. When I work with my friends on schoolwork, I tend to tell them “you are really good at this,” which is definitely not a growth mindset comment. I wanted to have real conversations with my students, but I was able to reframe them with positive compliments that don’t revolve around being smart or having natural talents. I hope to take these skills into my job next year as a teaching assistant to promote growth mindset attitudes.”* |
| *“Another part of the class that has really resonated with me is the impact of words on young children. We spoke about this influence a lot when it came to growth-mindset terms; not using the word “smart,” but maybe “hard-working” or “gritty” instead helps to teach children that intelligence isn’t an inherent valuable trait, but rather it is something that is proven based off of their work ethic and passion for a subject. This had made me reflect on how I see myself and my inherent worth because I realized that for a long time I believed my value came from how well I was doing in school. I felt proud when people would come up to me and call me “smart” and ask me questions because it felt like I was valued for this trait. However, this academic success did not come by itself; I had to work extremely hard to do well in school, and looking back on it I think the term “smart” minimized the amount of time and effort I put into my academics. Through this class I’ve realized that this probably took an unrealized toll on me, and I wanted to work to prevent that from happening to other girls during this experience. I really tried to stray away from these fixed-mindset terms in order to help these girls reach their full potential in the club. ”* |
| *“This class has ignited my passion for getting more involved within my community and has given me a real sense of purpose on this campus. This is something that I have struggled to find outside of academics so far in college”*  *“Over the course of the semester, I added another level to my learning objectives as we continued our work as a class and with the mentees. The added level was to accomplish my goals in fun and engaging ways to inspire the girls to look at science as something they could love and find success and a future”* |
| *“I learned the most about systemic barriers/other barriers in science and the importance of a growth mindset! I am likely to take this knowledge away and apply it to ensure any space I’m in is an inclusive one. I personally also have a very different perspective on what it means to be smart and how my identity is currently entangled with academic success and achievement (and I need to be kinder to myself!) This class has also shown me the importance of having these tough conversations and the ease of finding community with other students who want to support women and other minorities in STEM!”* |
| *“However, we quickly realized as a group that the science kits they provided gave too much structure for such a young group. We needed to use their energy and excitement to our advantage, and incorporate it towards science. As a result, I decided my learning objectives should be geared toward developing a general interest in science. I wanted the girls to become excited to take part in activities, think about what occurred, and craft up reasons for why it did. I wanted them to start asking questions, and test what would happen if they carried these out.*  *Instead of focusing on younger girls to build confidence in science-related fields, the most important aspect should be to create interest and excitement. For A, this seemed to be accomplished through challenging her, and making her think critically. This helped her build her confidence, as many times her guesses were right. This made her less nervous to be wrong, and when she was, she became even more interested in what truly occurred.”* |
| *“Once we had a more solid plan and brought our own materials and questions, I felt much more confidence in my abilities to get the girls interested and excited about science. When I acted more confidently, the girls paid more attention to me and were more likely to listen to what I had to say. Since we had planned ahead, it was easier to adapt the plan based on how the girls were acting and what they wanted to. One thing I focused on throughout all of this was having the girls make predictions, which became easier as I grew more comfortable with them and the activities.”* |

**Table S4:** Selected student statements from their *Learning Gains* essays are shown.

| **Student** | **Goals Identified in Instrumentality Essay** | **Learning Gain Reflections** |
| --- | --- | --- |
| 1 | *“I have always been passionate about serving others and partaking in community servic*e.”  *“The physical volunteering with the girls from the organization we will be partnering with, will provide me with valuable teaching and mentorship ski*lls” | *“I had no idea that I would meet so many wonderful people, learn more about myself and the world around me than I could have ever imagined, or be inspired to pursue my passion for science even further. I had no idea that as I was trying my hardest to get young girls interested in science, that they would teach me more about myself than I was teaching them.”* |
| 2 | *“I was extremely eager to take this course because of a realization that emerged following the Sarah Reisman talk last semester about diversity, equity, and inclusion in STEM.”*  *“Additionally, having to plan out my own lessons will help me with my communication skills and how I present information to others.”* | *“This year we have found a few major conclusions, STEM outreach to younger audiences need to revolve around fun. The stereotype kids gain where school, education, and homework isn’t fun is what kills any possibility of them listening to us “teach”. As soon as we bring science to them in a boring way, we lose their interest immediately. By creating fun, hands-on, interactive activities, the girls were more likely to listen to us and become curious about the science. Another conclusion is we have to know our audience and tailor our approach to them. Many of the girls were easily distracted, as soon as another girl was doing something, they wanted to as well. In my experience with my mentee, there was no way that she was going to pay attention to a 5 minute video, nevermind continue with a 7 week long project. I think I learned when I figured out a new game plan going forward. Lastly, these meetings and this course can (and will) be largely unpredictable. The incoming mentors have to be open minded, problem solvers who can connect with many different personalities.”* |
| 3 | *“The CBL portion of this class is an effort to promote STEM education to underrepresented students in the Worcester community. Institutions of injustice run deep in this country, and CBL won’t resolve these national issues, but this program strives to do good in our local area.”*  *“I want to get better at teaching through this program, specifically I am hoping to develop my communication skills.”* | *“One of my goals that I wrote about in the Instrumentality essay is to improve my communication skills. I was originally writing this as improving science communication skills to a general audience, but I believe that this has expanded over the course of the semester. Communicating accolades, capturing the attention of young students, and maintaining focus were all important skills that I learned in this course that go beyond the science aspect. Most importantly, communicating that the students were doing well through their effort and hardwork was a shift in how I approached this experience. When I work with my friends on schoolwork, I*  *tend to tell them “you are really good at this,” which is definitely not a growth mindset comment. I wanted to have real conversations with my students, but I was able to reframe them with positive compliments that don’t revolve around being smart or having natural talents. I hope to take these skills into my job next year as a teaching assistant to promote growth mindset attitudes.”* |
| 4 | *“I think understanding how students learn and the benefits of different methods of assessment and communicating with the students will be helpful with my personal goals of becoming a better teacher, especially when we read the education articles.”* | *“Another part of the class that has really resonated with me is the impact of words on young children. We spoke about this influence a lot when it came to growth-mindset terms; not using the word “smart,” but maybe “hard-working” or “gritty” instead helps to teach children that intelligence isn’t an inherent valuable trait, but rather it is something that is proven based off of their work ethic and passion for a subject. This had made me reflect on how I see myself and my inherent worth because I realized that for a long time I believed my value came from how well I was doing in school. I felt proud when people would come up to me and call me “smart” and ask me questions because it felt like I was valued for this trait. However, this academic success did not come by itself; I had to work extremely hard to do well in school, and looking back on it I think the term “smart” minimized the amount of time and effort I put into my academics. Through this class I’ve realized that this probably took an unrealized toll on me, and I wanted to work to prevent that from happening to other girls during this experience. I really tried to stray away from these fixed-mindset terms in order to help these girls reach their full potential in the club. ”* |
| 5 | *“I value and pursue CBL courses at Holy Cross as they allow me to reflect on personal*  *experiences and social aspects while giving back to the community in a positive and supportive manner.”*  *“As an individual, I can continue to gain valuable experience communicating with others.”* | *“This class has ignited my passion for getting more involved within my community and has given me a real sense of purpose on this campus. This is something that I have struggled to find outside of academics so far in college”*  *“Over the course of the semester, I added another level to my learning objectives as we continued our work as a class and with the mentees. The added level was to accomplish my goals in fun and engaging ways to inspire the girls to look at science as something they could love and find success and a future”* |
| 6 | *“I was interested in this course because I noticed the lack of “real life” opportunities for*  *STEM students, with all CBL courses falling under humanities or social sciences.”*  *“My other goal is to become a more effective mentor.”* | *“I learned the most about systemic barriers/other barriers in science and the importance of a growth mindset! I am likely to take this knowledge away and apply it to ensure any space I’m in is an inclusive one. I personally also have a very different perspective on what it means to be smart and how my identity is currently entangled with academic success and achievement (and I need to be kinder to myself!) This class has also shown me the importance of having these tough conversations and the ease of finding community with other students who want to support women and other minorities in STEM!”* |
| 7 | *“The chance to expand my academic mentoring in the Worcester community provides a new opportunity and environment to enhance my mentorship skills.”*  *“The discussions, reflections, and activities held during class-time will allow me to adequately prepare for my Girls Inc. experience. However, not everything goes as planned, and putting these ideas to use will allow me to think dynamically, adjusting my approaches to cater to the needs of the individual student.”* | *“However, we quickly realized as a group that the science kits they provided gave too much structure for such a young group. We needed to use their energy and excitement to our advantage, and incorporate it towards science. As a result, I decided my learning objectives should be geared toward developing a general interest in science. I wanted the girls to become excited to take part in activities, think about what occurred, and craft up reasons for why it did. I wanted them to start asking questions, and test what would happen if they carried these out.*  *Instead of focusing on younger girls to build confidence in science-related fields, the most important aspect should be to create interest and excitement. For A, this seemed to be accomplished through challenging her, and making her think critically. This helped her build her confidence, as many times her guesses were right. This made her less nervous to be wrong, and when she was, she became even more interested in what truly occurred.”* |
| 8 | *“I was interested in this course because I am interested in a career in teaching.”* | *“Once we had a more solid plan and brought our own materials and questions, I felt much more confidence in my abilities to get the girls interested and excited about science. When I acted more confidently, the girls paid more attention to me and were more likely to listen to what I had to say. Since we had planned ahead, it was easier to adapt the plan based on how the girls were acting and what they wanted to. One thing I focused on throughout all of this was having the girls make predictions, which became easier as I grew more comfortable with them and the activities.”* |

**Table S5:** Common thematic patterns of student Instrumentality Essays are highlighted: Yellow represents communication/mentoring skills; red represents community service, blue represents pedagogical skills, and green represents the desire to bridge community/service work with scientific disciplines. The third column includes excerpts from the Learning Gains Essay. It is evident that the students’ learning gains essays were much more complex and intersectional.

**Results from the Faculty-administered Survey**

The three-question survey administered by the faculty member prompted students to revisit the topics covered in the first learning block (training and preparation). The first question asked: “On a scale from 1 to 10, how confident do you feel about these concepts now as compared to the beginning of the semester?, i.e. how well can you explain these concepts to a friend now versus at the start of the semester?”. Student responses are tabulated in Table 6. On the whole, students reported an increase in their confidence on these topics.

| **Question 1:** On a scale from 1 to 10, how confident do you feel about these concepts now as compared to the beginning of the semester?, i.e. how well can you explain these concepts to a friend now versus at the start of the semester? | | | | | | | | | | | | | | | | | | | | |
| --- | --- | --- | --- | --- | --- | --- | --- | --- | --- | --- | --- | --- | --- | --- | --- | --- | --- | --- | --- | --- |
| **Concept** | **Confidence rating at semester start** | | | | | | | | | | **Confidence rating semester end** | | | | | | | | | |
|  | **1** | **2** | **3** | **4** | **5** | **6** | **7** | **8** | **9** | **10** | **1** | **2** | **3** | **4** | **5** | **6** | **7** | **8** | **9** | **10** |
| Bloom’s Taxonomy | 4 | 3 | 1 |  |  |  |  |  |  |  |  |  |  |  |  |  |  | 6 | 1 | 1 |
| Growth Mindset | 2 | 4 | 2 |  |  |  |  |  |  |  |  |  |  |  |  |  |  | 3 | 3 | 2 |
| Instrumentality | 4 | 4 |  |  |  |  |  |  |  |  |  |  |  |  |  |  |  | 5 | 1 | 2 |
| Implicit Biases | 1 | 2 | 5 |  |  |  |  |  |  |  |  |  |  |  |  |  |  | 2 | 4 | 2 |
| Misconceptions in Learning | 3 | 2 | 2 |  |  |  |  |  |  |  |  |  |  |  |  |  |  | 4 | 2 | 2 |
| Crafting Assessments | 2 | 3 | 3 |  |  |  |  |  |  |  |  |  |  |  |  |  |  | 2 | 3 | 3 |
| Creativity in STEM |  | 3 | 5 |  |  |  |  |  |  |  |  |  |  |  |  |  |  | 3 | 3 | 2 |

**Table S6:** Student responses to Faculty-administered Survey: Question 1. The table denotes how many students selected each rating.

The second question asked: “Which of the concepts in this course were the most valuable to you as a student? Select all that apply” Student responses are tabulated in Table 7. Unanimously, students reported that foundational pedagogical concepts (Bloom’s Taxonomy) as well as concepts on mindset (Growth Mindset and Implicit Bias) were the most valuable to their experiences.

| **Question 2:** Which of the concepts in this course were the most valuable to you as a student? Check all that apply. | |
| --- | --- |
| **Concept** | **Number of students that selected this concept** |
|  |  |
| Bloom’s Taxonomy | 8 |
| Growth Mindset | 8 |
| Instrumentality |  |
| Implicit Biases | 8 |
| Misconceptions in Learning |  |
| Crafting Assessments |  |
| Creativity in STEM |  |

**Table S7:** Student responses to Faculty-administered Survey: Question 2. The table denotes how many students selected each concept as being the most valuable to their learning.

The third question asked: “Would you like to share any other comments pertaining to the benefits of this course? Please limit responses to 1 – 2 sentences.” (Table 8).

| **Question 3:** Would you like to share any other comments pertaining to the benefits of this course? Please limit your responses to 1 – 2 sentences. | |
| --- | --- |
| **Student** | **Comment** |
| 1 | I never would have stopped to think about how I learn without this class. |
|  |  |
| 2 | I learned so much about how to actually connect with someone by talking to my mentee. |
| 3 | This class blew my mind. I’m going to tell all my friends that they need to take it. |
| 4 | Because of this class, I’m going to think so much more carefully about the words I use and the impact they have on people. |
| 5 | I wish I could take this class again! I’ve learned so much about who I am and where I belong. |
| 6 | I’m so glad we were able to take science out of the classroom! |
| 7 | This class made me so much more independent in how I learn. |
| 8 | I learned that learning is supposed to be messy and that’s OK! |

**Table S8:** Student responses to Faculty-administered Survey: Question 3. The table lists the student statements pertaining to the benefits of the course.

**Results from Re-taking Harvard IAT**

In the final assessment block, students re-took the Harvard IAT and reflected on any changes in their results. SI-Table 9 summarizes the initial to final IAT results across participants. At the beginning of the semester, the student responses fell into two categories: automatic association of male-science (ranging from strong to little/no), and automatic association of female-science (ranging from strong to moderate). The results are shaded to indicate the strength of the automatic association. A blue shade designates automatic association of male-science, and a green shade designates automatic association of female-science. Five students experienced a change in their biases. The fact that a change in bias is observed, even though the sample size is small, reinforces the extensive work in the literature that cognizance of bias is the first step to change the bias itself. (Ariely, D. The Honest Truth About Dishonesty; Harper Perennial, 2013.)

|  | **Bias at semester start** | **Bias at semester end** |
| --- | --- | --- |
| 1 | Moderate automatic association of male-science | Little to no automatic association of male-science |
| 2 | Little to no automatic association of male-science | Little to no automatic association of male-science |
| 3 | Moderate automatic association of *female-science* | Little to no automatic association of male-science |
| 4 | Slight automatic association of male-science | Slight automatic association of male-science |
| 5 | Strong automatic association of male-science | Slight automatic association of male-science |
| 6 | Moderate automatic association of male-science | Little to no automatic association of male-science |
| 7 | Slight automatic association of male-science | Strong automatic association of *female-science* |
| 8 | Slight automatic association of male-science | Slight automatic association of male-science |

**Table S9:** Initial to final IAT results across participants. Results show a change in 5 out of 8 students.

The five students that experienced a change in their biases referenced the following as possible reasons for these changes (SI-Table 10):

| **Student** | **Response** |
| --- | --- |
| 1 | Beginning to embrace their STEM identity |
| 3 | Significant personal experiences to challenge STEM identity |
| 5 | Extensive personal work to promote change |
| 6 | Internalization of growth mindset |
| 7 | Feeling of responsibility as a male scientist to be an agent of change |

**Table S10:** Students that experienced a change in their bias cited the reasons listed above.

The three students who did not experience a change, referenced (SI-Table 11):

| **Student** | **Response** |
| --- | --- |
| 2 | Not particularly focusing on changing their bias |
| 4 | Bias is so ingrained; it will take longer than a semester to change. |
| 8 | Bias is so ingrained; it will take longer than a semester to change. |

**Table S11:** Students that did not experience a change in bias cited the reasons listed above.
